# Activation of SIRT1/Nrf2/HO-1 and Beclin-1/AMPK/mTOR autophagy pathways by eprosartan ameliorates testicular dysfunction induced by testicular torsion in rats

**DOI:** 10.1038/s41598-024-62740-6

**Published:** 2024-05-31

**Authors:** Rania H. Abu-Baih, Dalia H. Abu-Baih, Sara Mohamed Naguib Abdel-Hafez, Moustafa Fathy

**Affiliations:** 1 Faculty of Pharmacy, Drug Information Center, Minia University, Minia, 61519 Egypt; 2 Department of Biochemistry and Molecular Biology, Faculty of Pharmacy, Deraya University, Minia, 61111 Egypt; 3 Deraya Center for Scientific Research, Deraya University, Minia, 61111 Egypt; 4 Department of Histology and Cell Biology, Faculty of Medicine, Minia University, Minia, 61519 Egypt; 5 Department of Biochemistry, Faculty of Pharmacy, Minia University, Minia, 61519 Egypt

**Keywords:** Eprosartan, Testicular torsion, Spermatogenesis, Autophagy, Apoptosis, mTOR, Biochemistry, Drug discovery, Molecular medicine

## Abstract

Testicular torsion carries the ominous prospect of inducing acute scrotal distress and the perilous consequence of testicular atrophy, necessitating immediate surgical intervention to reinstate vital testicular perfusion, notwithstanding the paradoxical detrimental impact of reperfusion. Although no drugs have secured approval for this urgent circumstance, antioxidants emerge as promising candidates. This study aspires to illustrate the influence of eprosartan, an AT1R antagonist, on testicular torsion in rats. Wistar albino rats were meticulously separated into five groups, (n = 6): sham group, eprosartan group, testicular torsion-detorsion (T/D) group, and two groups of T/D treated with two oral doses of eprosartan (30 or 60 mg/kg). Serum testosterone, sperm analysis and histopathological examination were done to evaluate spermatogenesis. Oxidative stress markers were assessed. Bax, BCL-2, SIRT1, Nrf2, HO-1 besides cleaved caspase-3 testicular contents were estimated using ELISA or qRT-PCR. As autophagy markers, SQSTM-1/p62, Beclin-1, mTOR and AMPK were investigated. Our findings highlight that eprosartan effectively improved serum testosterone levels, testicular weight, and sperm count/motility/viability, while mitigating histological irregularities and sperm abnormalities induced by T/D. This recovery in testicular function was underpinned by the activation of the cytoprotective SIRT1/Nrf2/HO-1 axis, which curtailed testicular oxidative stress, indicated by lowering the MDA content and increasing GSH content. In terms of apoptosis, eprosartan effectively countered apoptotic processes by decreasing cleaved caspase-3 content, suppressing *Bax* and stimulating *Bcl-2* gene expression. Simultaneously, it reactivated impaired autophagy by increasing Beclin-1 expression, decreasing the expression of SQSTM-1/p62 and modulate the phosphorylation of AMPK and mTOR proteins. Eprosartan hold promise for managing testicular dysfunction arising from testicular torsion exerting antioxidant, pro-autophagic and anti-apoptotic effect via the activation of SIRT1/Nrf2/HO-1 as well as Beclin-1/AMPK/mTOR pathways.

## Introduction

Testicular torsion, an acute scrotal ailment, initiated by torsional rotation of the testis around its longitudinal axis. Untimely recognition bears the ominous potential to precipitate ischemic insults and subsequent testicular atrophy. Yet, a glimmer of hope exists when surgical intervention is orchestrated within six hours, the majority of testicles can be salvaged from the precipice of irreversible loss^1–3^. Testicular torsion affects one out of every four thousand below the age of 25, with testicular torsion ranging from 25 to 50%, of all instances of acute scrotal conditions^4–6^. It can manifest across the lifespan, yet it predominantly exhibits a bimodal pattern^7,8^. The extent of testicular torsion's temporal persistence significantly influences the likelihood of consequential testicular dysfunction and impaired spermatogenesis, which, in turn, may precipitate male infertility^9^. The resumption of blood flow to previously ischemic tissues during detorsion leads to an exacerbated release of reactive oxygen species (ROS)^10^ culminating in the diminishment of cellular viability, as a consequence of lipid peroxidation, protein denaturation, and DNA damage^11,12^.

Silent information regulator transcript-1 (SIRT1) is a NAD + dependent histone deacetylase that is intimately associated with some biological processes as apoptosis, inflammation, and oxidative stress^13^. SIRT1 exhibits the ability to modulate significant transcription factors like nuclear erythroid factor 2-related factor 2 (Nrf2) and heme oxygenase-1 (HO-1). These factors assume a pivotal role in orchestrating antioxidant, anti-inflammatory, and cytoprotecting responses^14^.

Autophagy is perceived as a cellular defense mechanism aimed to eliminate impaired mitochondria and improperly folded proteins via the autophagosome-lysosome pathway^15,16^. In instances of cellular distress, autophagy is considered as a pro-survival mechanism, effectively averting apoptotic cell demise and upholding the equilibrium of cellular homeostasis^17,18^. Under typical physiological conditions, autophagy operates within constrained parameters. Nevertheless, discernible stimuli, encompassing starvation, ischemia/hypoxia, and infection have been recognized as potent instigators of autophagic responses. The process of autophagy flux encompasses a series of sequential events, commencing with the initiation of phagophore formation and sequestration of impaired organelles. As such, the activation of autophagy is deemed pivotal in ameliorating ischemia reperfusion injury (IRI)^19,20^.

It is worth highlighting that sequestome 1/protein 62 (SQSTM-1/p62) besides Beclin-1 have essential role in autophagic processes^21,22^. SQSTM-1/p62 facilitates ubiquitinylated proteins’ removal^23^. Whilst Beclin-1 assumes a pivotal role in the autophagosomes generation during the autophagic sequestration process^24,25^. Of note, the orchestration of autophagic processes is influenced by the activation of the 5’ adenosine monophosphate-activated protein kinase (AMPK) and modulation of the mammalian target of rapamycin (mTOR) pathway^25,26^. Intriguingly, prior research has elucidated that IRI in rodent models can be partially alleviated through the AMPK/mTOR pathway stimulation^27^.

To uphold tissue equilibrium, the intricate regulation of autophagy and apoptosis is imperative^17,18^. Autophagy, acting as a resilience-promoting mechanism, enhance cells’ viability. Conversely, apoptosis is a fatality-predisposing process, selectively eliminating severely impaired cells^15,18^. The interplay between autophagy and apoptosis has been extensively chronicled in diverse testicular diseases^28^. In this context, autophagy has proved its ability to exert an inhibitory influence on apoptotic cell demise^28^, as well as in various experimental scenarios encompassing testicular IRI^29^, neurological diseases^30^ and hepatic injuries^31^.

The testis possesses its own localized renin-angiotensin system (RAS) that functions in the regulation of testicular blood flow besides overall testicular function^32^. Angiotensin II, the principal mediator molecule of the RAS, can potentially contribute to IRI by inducing vasoconstriction, oxidative stress, inflammation, and tissue remodeling^32^. Recent studies have indicated that drugs which modulate RAS may hold significant promise in conferring protective benefits to several organs, encompassing heart, kidneys, brain, and liver^33^. Eprosartan, an AT1R antagonist, is used frequently in the management of hypertension^34–36^. Eprosartan has been empirically established to wield anti-inflammatory properties via mitigating inflammatory cytokine expression and oxidative stress within the vascular endothelium^37–39^. In light of these deliberations, this study, for the first time, aimed to elucidate, on molecular bases, the possible ameliorative impact of eprosartan in testicular dysfunction evoked by testicular torsion in rats.

## Materials and methods

### Drugs and chemicals

Eprosartan was provided as a gift from Hochster Pharmaceutical Industries, Cairo, Egypt. Eprosartan was dissolved in 1% aqueous solution of carboxymethylcellulose (CMC).

### Animals

Adult male Wistar albino rats, displaying weights within the range of 250–300 g and aged between 6 and 8 weeks, were meticulously procured from the Animal Facility Center at Minia University. Animals were diligently housed in accordance with the stringent standards of good laboratory practice, ensuring a controlled environment featuring a temperature range of 20–24 °C, a relative humidity of 55% ± 10%, precise lighting regulation, and a meticulously maintained 12-h light–dark cycle. Animals were accommodated under standard housing conditions, ensconced in cages, and provided with chow and tap water ad libitum for a duration of one week, allowing them to acclimate prior to commencing the experiment. The current study was carried out under the auspices of the Faculty of Pharmacy at Minia University, Egypt. The animals had a 12-h fasting period before the initiation of the study.

The experiments were performed regarding ARRIVE guidelines and all methods were conducted in accordance with the relevant regulations and guidelines of the ethical review board of Faculty of Pharmacy at Minia University. The experimental protocols were approved by The Research Ethics Committee, Faculty of Pharmacy, Minia University, Minia, Egypt (approval number: 230901).

### Experimental testicular torsion/detorsion (T/D) procedures

All surgical procedures were meticulously executed within the confines of stringent aseptic protocols. The surgical operation was meticulously conducted in strict accordance with antecedent experimental investigations^40–42^. Briefly, rats were fixed on the table in a supine position, with their ventral side facing upwards. Subsequently, each rat underwent depilation at the ilioinguinal region, followed by the application of a 10% povidone iodine solution for disinfection. Thereafter, an intraperitoneal injection of anesthesia (ketamine 100 mg/kg and xylazine 10 mg/kg) was administered. Via a longitudinal scrotal incision, the left testis was unveiled, where it was deftly rotated clockwise through an exacting angle of 720 degrees to induce torsion and secured in this state by clipping. Afterwards, the scrotum was shrouded with a meticulously prepared swathe of cotton imbued with normal saline. One hour later, the testicular torsion was expertly reversed, gently restoring the testis to its natural position within the scrotum, thereby facilitating a reperfusion period spanning four hours. Then, the scrotal incision was sealed with 2/0 silk suture. Eprosartan was dissolved in CMC^38,43^ and was administered orally one hour before T/D in low and high doses to determine whether escalating the dosage could yield more favorable outcomes.

### Experimental groups

**Figure 1:**
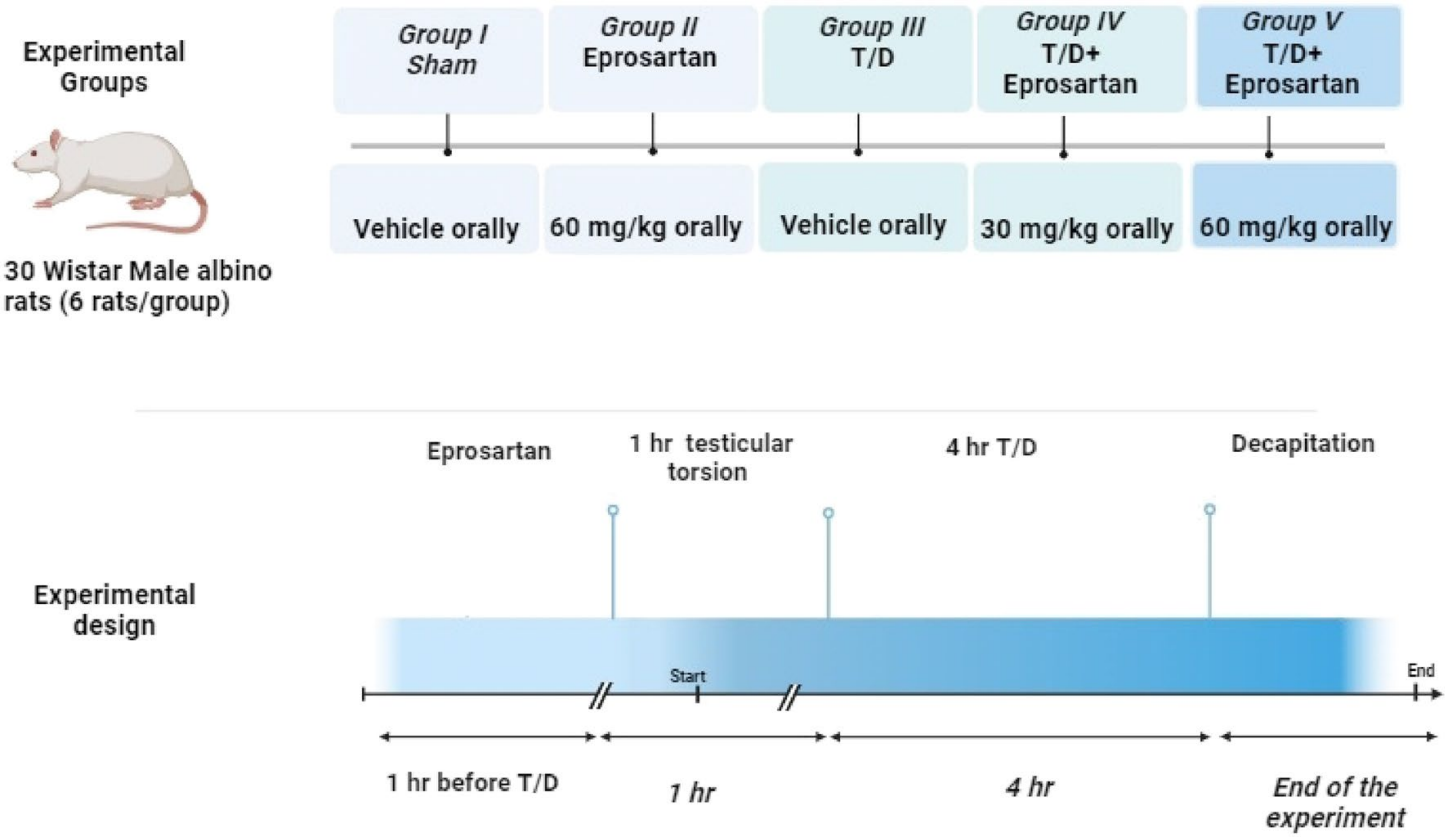
Schematic depiction of the experimental design, illustrating the various study groups and the temporal aspects of torsion and detorsion. T/D; torsion/detorsion.

Rats were haphazardly split up into five groups, as shown in Fig. 1:

- Group I (Sham control group): Animals received vehicle (1 ml orally) and were subjected to all operational procedures with the exception of vascular occlusion.
- Group II (Eprosartan 60 mg/kg): Rats received eprosartan (60 mg/kg) once orally^38^ and animals were subjected to all operational procedures with the exception of vascular occlusion.
- Group III (T/D): Testicular torsion induced for 1 h, then detorsion done and sustained for 4 h on the left testis.
- Group IV (T/D + Eprosartan 30 mg/kg): In this group, rats were given eprosartan (30 mg/kg, orally) 1 h before T/D, then 1 h testicular torsion was induced in the left testis, and subsequently detorsion done and sustained 4 h^44,45^.
- Group V: (T/D + Eprosartan 60 mg/kg): in this group, rats were given eprosartan (60 mg/kg, orally) 1 h before T/D, then 1 h testicular torsion was induced in the left testis, and subsequently detorsion done and sustained 4 h^38^. The time of torsion and detorsion was established based on previously described^46^.

### Sample preparation and tissue isolation

At the end of the experiment, the rats were individually weighed, intraperitoneally injected with thiopental (50 mg/kg), and then humanely sacrificed by decapitation. Blood was drawn from the jugular vein and centrifuged at 5000 rpm for 15 min to separate the serum then, frozen until further analysis. Testes were excised, washed with saline, and weighed. Rapid dissection of the testes was done. Two sections of testicular tissues were collected. The first section was rapidly stored at a temperature of − 80 °C for subsequent biochemical analysis. While the other section was immersed in Bouin’s solution for fixation (50% saturated picric acid, 35% distilled water, 10% formalin and 5% acetic acid) for histological examinations. Finally, the cauda epididymis was gathered and incised, and the seminal fluid was promptly collected to evaluate the characteristics of the sperm.

### Examination of sperm characteristics

#### Assessment of sperm count

The evaluation of sperm in the epididymis was conducted utilizing established conventional techniques^47^. The excised cauda epididymis of rats was acquired and subjected to precise incisions using sterile scissors before being transferred to a 1 mL of preheated phosphate buffer saline (PBS). Delicate agitation was done to stimulate the release of spermatozoa from the dissected tissue into the warmed PBS^48^. Subsequently, each sample was incubated at a temperature of 37 °C for a duration of 20 min. Then, a hemocytometer was utilized to enumerate the spermatozoa using a light microscope (at 400× magnification).

#### Assessment of sperm motility

Sperm motility was assessed by introducing a single droplet of 2.9% sodium citrate, preheated to a temperature of 37 °C, into the seminal fluid. In a timeframe of 2 to 4 min, the sperm suspension (50 µL) was carefully applied onto a glass slide. Subsequently, the spermatozoa motility was assessed by observing their movement in ten microscopic fields. Here, the percentage of motile sperm was assessed in relation to the total sperm number^49^.

#### Assessment of sperm viability and morphological abnormalities

To assess the vitality of sperm and detect morphological defects, a single droplet of the epididymal content was mixed with one droplet of Eosin-Negrosin stain. A total of 300 spermatozoa were subjected to random inspection at a magnification of 400×, and the percentage of viable spermatozoa was detected^49^.

In respect to sperm morphological abnormalities, the overall percentage of sperm abnormalities was assessed in accordance with the methodology outlined in a previous study^50^. Regarding this matter, the observed sperm abnormalities encompassed irregularities in both the structure of the sperm head and the tail.

### Histopathological examination

Each animal’s testis was fixed in Bouin’s solution, dehydrated in increasing grades of alcohol, cleaned with xylene, promptly embedded in paraffin wax, and sectioned at 5 m thick^51^. Light microscope (Olympus CX23LEDRFS1, Tokyo, Japan) was used to examine the sections to investigate the histological alterations. The observer was blinded to the group identity of each slide. Screening of sections was conducted under light microscope magnification ×200, and ×400.

Morphometric analysis utilizing an image analysis system (Leica Q500IW, Leica Microsystems, Wetzlar, Germany) and software (Leica QWIN standard version 2.3, Leica Microsystems) was done for semi quantitation of histological alterations and estimation of spermatogenesis on twenty seminiferous tubules across different experimental groups using cosentino^52^ and Johnsen^53^ scores respectively. Scoring was shown in Tables 1 and 2. Scoring was done in hematoxylin and eosin (H&E)-stained slides in eight non-overlapping fields from each rat of all groups, using Image J (version: 0.9.0, freeware; rsbweb.nih.gov/ij).

**Table 1:** Histopathological grading score (Cosentino’s score).

Grade	Characteristics
I	Normal testicular morphology
II	Few orderly germinal cells with closely crowded seminiferous tubules
III	Anarchic sloughed germinal cells, with a reduction in pyknotic nuclei size and few distinct seminiferous tubule borders
IV	Closely packed seminiferous tubules and coagulative necrosis of the germinal cells

Semi quantitation of histological alterations on different seminiferous tubules in the whole groups by grading the tubules between I & IV, and the mean scores were obtained by dividing the totality to the whole number of testicular tubules.

**Table 2:** Spermatogenesis grading system (Johnsen’s scoring system).

Score	Morphological picture
10	Normal spermatogenesis
9	Minimum defect in spermatogenesis with multiple late spermatids, little disorganized epithelium
8	Less than five spermatozoa/tubule, less late spermatids
7	No spermatozoa, absent late spermatids, many early spermatids
6	No spermatozoa, absent late spermatids with little early spermatids
5	No spermatozoa or spermatids, many spermatocytes
4	No spermatozoa or spermatids, fewer spermatocytes
3	Only spermatogonia
2	No germinal cells, Sertoli cells only
1	No seminiferous epithelium

By using Johnsen’s scoring system, the impact of ischemia on the spermatogenesis can be detected. The grades were from 1 to 10, and the total scores were obtained via dividing to the whole number of testicular tubules.

### Biochemical analysis

#### Assessment of serum testosterone

Serum testosterone level was assessed via testosterone ELISA kit (CUSABIO, Houston, Texas, USA, Cat. no. CSB-E05100r) regarding manufacturer’s instructions.

#### Assessment of testicular oxidative stress parameters

The detection of testicular malondialdehyde (MDA) (Bio-Diagnostic, Giza, Egypt) was performed using the method previously reported. In summary, the use of thiobarbituric acid in conjunction with MDA leads to the formation of pink-colored thiobarbituric acid reactive species. A calorimetric technique was used to determine the color's intensity at 534 nm, and it was inversely correlated with the MDA concentration. The MDA levels were measured and expressed as nmol/g tissue^54^.

The determination of testicular reduced glutathione (GSH) was conducted utilizing a commercially available spectrophotometric kit (Biodiagnostic, Cairo, Egypt). The thiol moiety present in glutathione exhibits a reduction reaction with Ellman's reagent, also known as 5,5-dithio-bis-2-nitrobenzoic acid leading to a yellow-colored compound (5-thio-2-nitrobenzoic acid), which was quantified spectrophotometrically at 412 nm^55^.

### Enzyme-linked immunosorbent assay (ELISA) analysis

Rat-specific ELISA kits for SIRT1 (Elabscience, Texas, USA, Cat. no. E-EL-R1102), Nrf2 (NOVUS BIOLOGICALS, Texas, USA, Cat. no. NBP3-08161), and HO-1 (ELISAGenie, Dublin, Ireland, Cat. no. RTFI00859) were utilized for the assay of each respective target.

The quantification of Beclin-1 and SQSTM-1/p62 levels in testicular tissue homogenates was performed using ELISA kits (CUSABIO, Houston, Texas, USA, Cat. no. CSB-EL002658RA) and (SunLong Biotech Co., LTD, Hangzhou, Zhejiang, China, Cat. no. SL1363Ra), respectively regarding the guidelines provided by the manufacturer.

### Western blotting analysis

The evaluation of p-AMPK, t-AMPK, p-mTOR and t-mTOR expressions in testicular tissues was conducted through the utilization of western blot analysis. In order to quantify the protein concentration, we utilized a Bradford Protein Assay Kit (SK3041; Markham, Ontario, L3R 8T4 Canada). Each sample was loaded with a total of 50 µg of protein and subjected to electrophoresis on a polyacrylamide gel, followed by transfer onto a PVDF membrane. Tween 20 (TBS-T) buffer and 3% bovine serum albumin (BSA) were employed to perform membrane blocking at a temperature of 25 °C for a duration of one hour. Primary antibodies for β-actin (Santa Cruz Biotechnology, Santa Cruz, CA), Rabbit Anti-p-AMPK alpha 1 (phospho T183) (1:1000, ab133448, Abcam, UK), Rabbit Anti-AMPK alpha 1 antibody (1:1000, ab32047, Abcam, UK), Rabbit Anti-p-mTOR (phospho S2448) antibody (1:1000 ab109268, Abcam, UK) and Rabbit Anti-mTOR antibody (1:1000 ab134903, Abcam, UK) were purchased. Overnight incubation was conducted in individual primary antibody solutions targeting the blotted target protein, maintained at a temperature of 4 °C. Following the washing procedure, the blot was subjected to incubation with a goat anti-rabbit polyclonal immunoglobulin coupled with horseradish peroxidase (1:5000) obtained from Cell Signaling Technology Inc., Massachusetts, USA. Through the process of antibody interaction, the detection of a chemiluminescence substrate (specifically, ClarityTM Western ECL substrate, Bio-Rad cat#170-5060) was achieved. The chemiluminescent signals were recorded utilizing an imager based on a CCD camera. The band intensity of targeted proteins was assessed using Image J software (version: 0.9.0, freeware; rsbweb.nih.gov/ij), which allowed for the measurement of protein levels relative to the control sample β actin. This analysis was performed using the ChemiDoc MP imager.

### Quantitative real-time polymerase chain reaction (qRT-PCR) analysis

Total RNA extraction was done via Trizol reagent (Invitrogen, Waltham, MA, USA) regarding the guidelines stipulated by the manufacturer. This procedure aimed to evaluate the expression of mRNA for the target genes. The extracted RNA’s concentration was estimated utilizing the Nanodrop 1000 instrument (Thermo Scientific, Waltham, Massachusetts, USA). The cDNA synthesis process was initiated by employing the High-capacity cDNA Reverse Transcription Kit. The Maxima SYBR green master mix, manufactured by thermo-scientific (Waltham, MA, USA) was employed in a real-time PCR experiment to quantify the total mRNA of targeted genes. The step one real-time PCR platform was utilized for this quantitative analysis^56^. The target gene primer sequences were acquired by consulting the National Centre for Biotechnology Information (NCBI) (Table 3). The quantification of the target genes' expression was conducted utilizing the 2^−ΔΔCt^ methodology, which encompassed the normalization of the obtained data with respect to the *glyceraldehyde-3-phosphate dehydrogenase* (*GAPDH*), commonly referred to as the housekeeping gene.

**Table 3:** Primer sequences.

Genes	Primer sequence
*Bax*	Forward: 5′-CACGTCTGCGGGGAGTCAC-3′
	Reverse: 5′-TAGAAAAGGGCAACCACCCG-3′
*Bcl-2*	Forward: 5′-GGGCTACGAGTGGGATACTG-3′
	Reverse: 5′-GACCCCACCGAACTCAAAGA-3′
*GAPDH*	Forward: 5′-CTCTCTGCTCCTCCCTGTTC-3′
	Reverse: 5′-CGACATACTCAGCACCAGCA-3′

### Statistical analysis

For each group, the values are displayed as means ± standard deviation (SD) (n = 6). The one-way analysis of variance and Tukey's multiple comparisons test were employed to examine the study’s data. These statistical tests were conducted utilizing GraphPad Prism software, version 5 (Inc., San Diego, USA). When the *p* value is lower than 0.05, the difference was deemed statistically significant.

### Ethical approval

The experiments were performed regarding ARRIVE guidelines and all methods were conducted in accordance with the relevant regulations and guidelines of the ethical review board of Faculty of Pharmacy at Minia University. The experimental protocols were approved by The Research Ethics Committee, Faculty of Pharmacy, Minia University, Minia, Egypt (approval number: 230901).

## Results

### Effect of eprosartan on sperm characteristics

**Figure 2:**
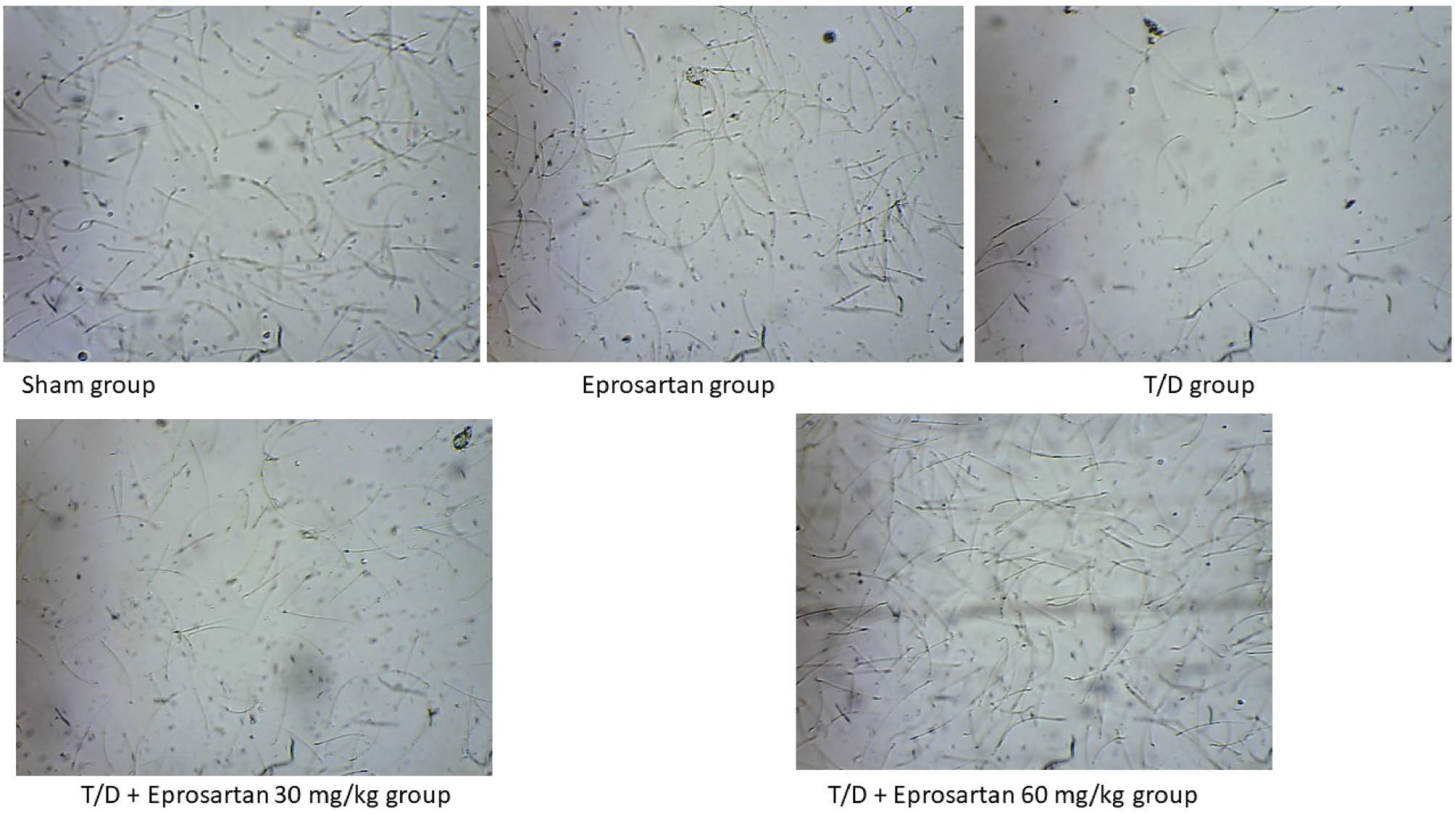
Representative microscopic field of semen analysis for all study groups. Images were taken by Dr. Dalia H. Abu-Baih, Department of Biochemistry and Molecular Biology, Faculty of Pharmacy, Deraya University, Minia 61111, Egypt; dalia.hamdy@deraya.edu.eg.

The scrutiny of testicular spermatogenesis was executed by an assessment of diverse sperm parameters, encompassing sperm count, motility, viability, and abnormalities. Related to the sham group, the rats subjected to T/D exhibited a substantial diminish in sperm count (*p* < 0.05) to 21.31 million/ml, a profound decrease in motility (*p* < 0.05) to 41.12%, and a significant dip in viability (*p* < 0.05) to 41.75%. Furthermore, they displayed a notable escalation in sperm abnormalities (*p* < 0.05) to 28.64%, as shown in Table 4 and Fig. 2. While eprosartan treatment at a dosage of 30 mg/kg effectively counteracted these disruptions, yielding significant improvements comparing to T/D, notably in sperm count (*p* < 0.05), recording an increase of 27.05%, motility (*p* < 0.05), surging by 46.59%, and viability (*p* < 0.05), soaring by 54.82%. Additionally, eprosartan (30 mg/kg) exhibited its prowess by significantly curtailing sperm abnormalities (*p* < 0.05), reducing them to 21.38% compared to T/D rats. Meanwhile, eprosartan at a dosage of 60 mg/kg yielded remarkable results compared to T/D rats (*p* < 0.05), with a substantial elevation in sperm count to 31.10%, a notable surge in motility to 67.93%, and a remarkable boost in viability by a staggering 79.63%. Furthermore, it achieved a significant diminish in sperm abnormalities to 11.60%. Astonishingly, the high dose of eprosartan exhibited markedly superior results in terms of sperm count, motility, viability, and a notable diminish in sperm abnormalities related to the low-dose eprosartan-treated group (*p* < 0.05). These compelling findings underscore eprosartan's potential in ameliorating disrupted spermatogenesis in T/D rats.

**Table 4:** Impact of eprosartan on sperm count, motility, viability and morphological abnormalities.

Groups	Sperm count (million/ml)	Sperm motility (%)	Sperm viability (%)	Sperm abnormality (%)
Sham	42.81 ± 0.51	74.86 ± 0.70	78.49 ± 2.80	12.74 ± 1.17
Eprosartan	41.46 ± 2.05	74.53 ± 0.98	78.33 ± 2.28	12.44 ± 0.79
T/D	21.31 ± 0.60*	41.12 ± 0.86*	41.75 ± 1.32*	28.64 ± 1.60*
T/D + Eprosartan 30	27.05 ± 1.17*^, #^	46.59 ± 0.90*^, #^	54.82 ± 2.55*^, #^	21.38 ± 2.08*^, #^
T/D + Eprosartan 60	31.10 ± 2.10*^, #, $^	67.93 ± 1.16*^, #, $^	79.63 ± 2.64*^, #, $^	11.60 ± 1.38^#, $^

Statistical analyses were performed employing one-way ANOVA followed by Tukey’s post hoc test, n = 6, mean ± SD. *,#,$; *p* < 0.05 versus sham, T/D, or T/D + eprosartan 30, respectively.

### Histopathological analysis

**Figure 3:**
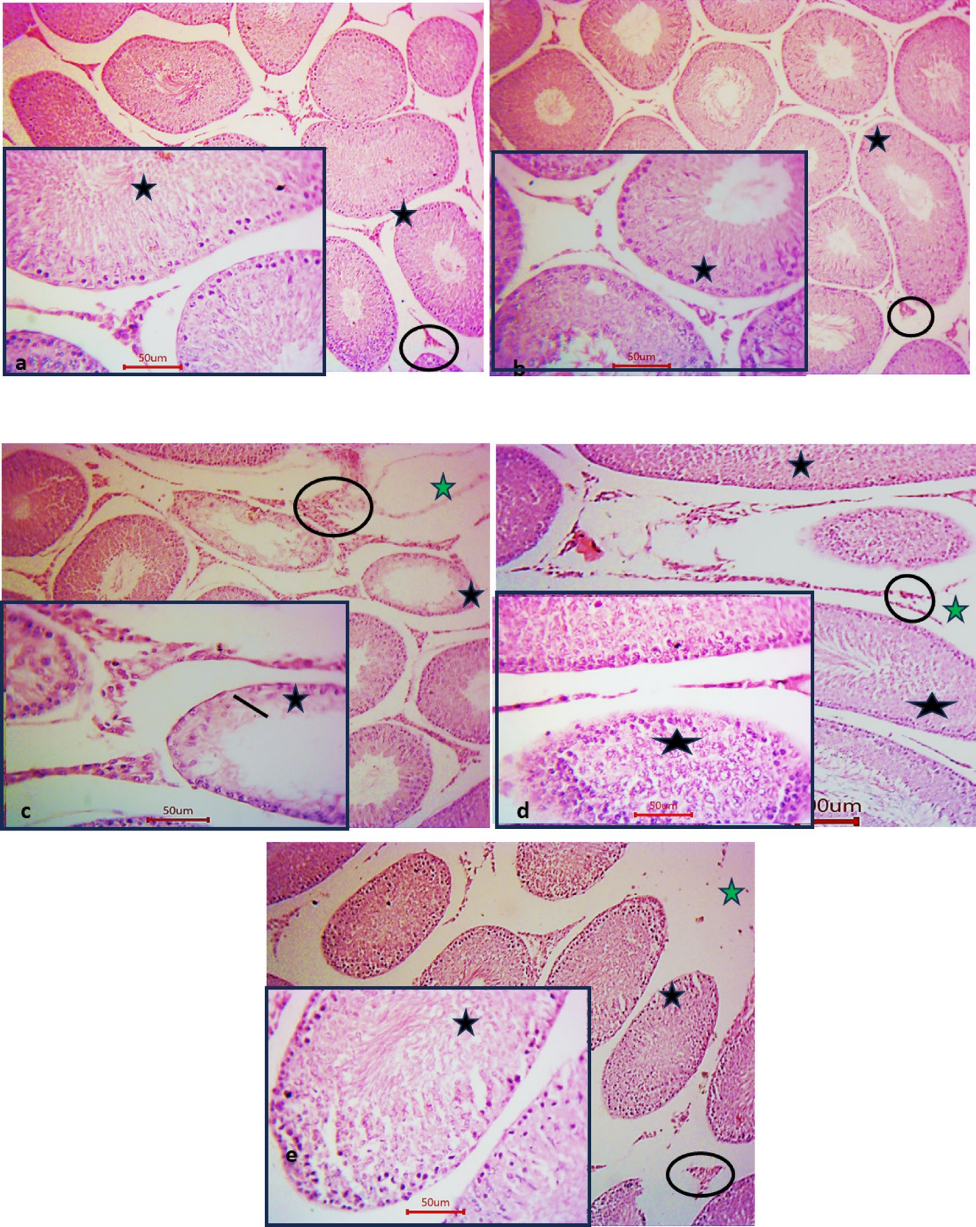
Photomicrographs of rat’s testicular sections. (**a** & **b**) represent sham and eprosartan groups, respectively, displaying the same histological structure. The seminiferous tubules (black star) are noticed lined with germinal epithelium. The interstitial cells of Leydig (circles) are noticed lying between these tubules. (**c**) represents T/D group showing distorted seminiferous tubules (black star). Notice the reduction in germinal cells (line) and the widened interstitium (green star). Distorted interstitial cells of Leydig (circles) is also noticed. (**d**) represents T/D + Eprosartan 30 group showing focal distorted seminiferous tubules (black star) but interstitial cells of Leydig appear more or less normal. Widing interstitial (green arrow) is noticed. (**e**) represents T/D + Eprosartan 60 group showing apparent normalization of the seminiferous tubules and interstitial cells of Leydig (circle), but still widing interstitial (green arrow) is noticed. Hematoxylin and eosin; Scale bar = 100; insets = 50.

**Figure 4:**
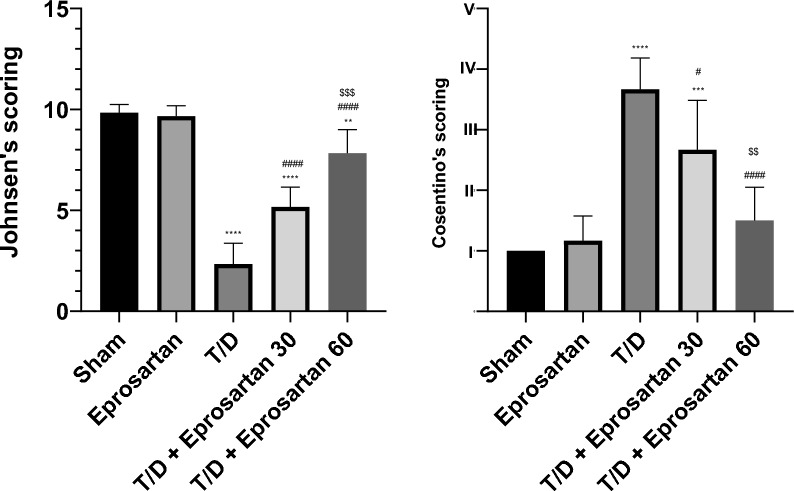
The effect of eprosartan on testicular histopathology and spermatogenic indices in all experimental groups, Statistical analyses were performed employing one-way ANOVA followed by Tukey’s post hoc test, n = 6, mean ± SD, where ****; *p* < 0.0001, ***; *p* < 0.001, **; *p* < 0.01, compared to the sham group, and #### *p* < 0.0001, # *p* < 0.05 compared to the T/D group, $$$; *p* < 0.001, $$; *p* < 0.01, compared to the T/D + eprosartan 30.

H&E sections of groups I & II (sham and eprosartan rats) showed the same morphological structure. The testis was formed of seminiferous tubules of variable size lined with germinal epithelium. The interstitial cells of Leydig were noticed lying between these tubules. Meanwhile photomicrographs of rat’s testicular section of group III (T/D rats) displayed distorted seminiferous tubules. This distortion was in the form of reduction of their germinal cells. Distorted interstitial cells of Leydig and widened interstitial were clearly noticed among the sections. Furthermore, group IV (T/D + Eprosartan 30 group) displayed focal distorted seminiferous tubules but interstitial cells of Leydig appear more or less normal. Widing interstitial was also noticed. While group V (T/D + Eprosartan 60 group) showed an apparent normalization of the seminiferous tubules and interstitial cells of Leydig, but still widing interstitial was noticed, as seen in Figs. 3 and 4.

### Influence of eprosartan treatment on the relative testicular weight and serum testosterone level

To explore the impact of T/D on the wight of the testis, we assessed the relative testicular weight of all animals across various experimental groups. As illustrated in Table 5, T/D precipitated a notable and statistically significant reduction (*p* < 0.0001) in relative testicular weight compared to the sham group. Our results unveiled that eprosartan at low and high doses produced significant restoration of testicular weight in a dose-dependent way in contrast to the testicular T/D group (*p* < 0.0001). Notably, the disparity between the sham group and the eprosartan-treated one did not attain statistical significance. Nevertheless, a noteworthy differentiation emerged when comparing the effects of the low and high doses of eprosartan (*p* < 0.01).

**Table 5:** Impact of eprosartan on relative testicular weight and serum testosterone level.

Groups	Relative testicular weight (g)	Serum testosterone (ng/ml)
Sham	0.75	2.58
Eprosartan	0.71	2.57
T/D	0.49****	0.34****
T/D + Eprosartan 30 mg/kg	0.67****^, ####^	2.21****^, ####^
T/D + Eprosartan 60 mg/kg	0.72^####, $$^	2.43^####, $$^

Data are presented as mean ± SD of 6 replicates; ****p < 0.0001 versus sham, ####*p* < 0.0001 versus T/D, $$*p* < 0.01 versus T/D + Eprosartan 30 mg/kg.

Additionally, the testicular T/D precipitated a marked reduction (*p* < 0.0001) in serum testosterone levels, when contrasted with the sham group. Intriguingly, our results revealed that eprosartan at both doses induced a substantial (*p* < 0.0001) reversal of the testosterone levels in a dose-dependent manner as compared to the T/D group. While the disparity between the sham and eprosartan-administered groups did not reach a level of statistical significance, a conspicuously significant differentiation did manifest when contrasting the low and high doses of eprosartan (*p* < 0.01).

### Influence of eprosartan treatment on oxidative stress parameters

**Figure 5:**
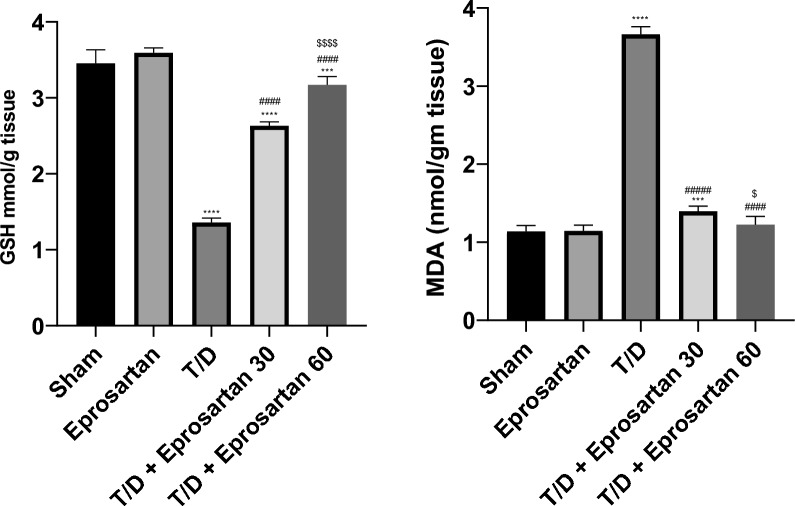
Eprosartan ameliorates testicular T/D-induced oxidative stress. Effect of eprosartan on testicular GSH and MDA levels across all experimental groups. Statistical analyses were performed employing one-way ANOVA followed by Tukey’s post hoc test, n = 6, mean ± SD. where ****; *p* < 0.0001, ***; *p* > 0.001, compared to the sham group, ####; *p* < 0.0001, compared to the T/D group, *p* < 0.0001, $; *p* < 0.05, compared to the T/D + eprosartan 30 group.

The influence of eprosartan on the testicular redox changes was studied by determining the antioxidant status alongside the MDA levels. Related to the sham group, testicular T/D caused substantial (*p* < 0.0001) decrease in GSH level to 1.35 mmol/g alongside a substantial (*p* < 0.0001) elevation in MDA to 3.66 nmol/g, as shown in Fig. 5. Meanwhile, administration of eprosartan (30 or 60 mg/kg) evinced a substantial elevation (*p* < 0.0001) in the level of GSH to 2.63 mmol/g and 3.17 mmol/g, respectively compared to T/D group. In a strikingly dose-dependent fashion, this intervention also brought about a notable reduction (*p* < 0.0001) in testicular MDA levels, diminishing them to 1.39 nmol/g and 1.22 nmol/g, respectively, as compared to the T/D group. No statistical significance delineated itself between the sham and eprosartan group, whereas a conspicuous distinction did emerge between the treatment with the lower and higher doses of eprosartan (*p* < 0.0001 for GSH and *p* < 0.05 for MDA).

### Eprosartan activates the SIRT1/Nrf2/HO-1 axis in testicular T/D rats

**Figure 6:**
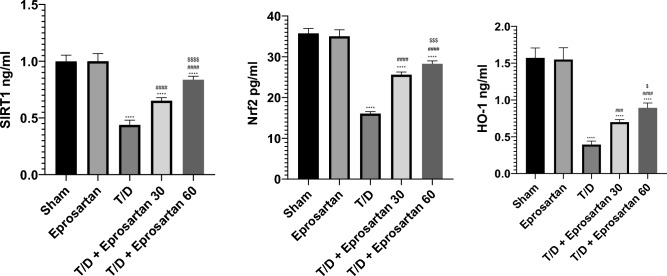
Eprosartan suppressed testicular SIRT1, Nrf2 and HO-1 proteins expression, across all experimental groups. Statistical analyses were performed employing one-way ANOVA followed by Tukey’s post hoc test, n = 6, mean ± SD. where ****; *p* < 0.0001, compared to the sham group, ####; *p* < 0.0001, ###; *p* < 0.001, compared to the T/D, $$$$; *p* < 0.0001, $$$; *p* < 0.001, $; *p* < 0.05, compared to T/D + eprosartan 30 group.

The influence of eprosartan on the testicular redox alterations precipitated by T/D was evaluated by assessing the alterations in the SIRT1/Nrf2/HO-1 cascade. Related to sham group, the testicular tissues of T/D rats exhibited marked exacerbation of oxidative disturbances. This was strikingly evident through a substantial decrement (*p* < 0.001) in SIRT1, Nrf2, and HO-1 testicular contents, to 0.43 ng/ml, 16.06 pg/ml, and 0.39 ng/ml, respectively, related to sham group as delineated in Fig. 6. Concurrently, eprosartan treatment at a dosage of 30 mg/kg elicited a noteworthy augmentation in the SIRT1 (*p* < 0.0001), Nrf2 (*p* < 0.0001), and HO-1 (*p* < 0.001) testicular contents, registering increases to 0.65 ng/ml, 25.61 pg/ml, and 0.69 ng/ml, respectively, when compared with the T/D group. Notably, the administration of eprosartan at a dose of 60 mg/kg exhibited a significantly more pronounced upregulation (*p* < 0.0001) in the testicular contents of SIRT1, Nrf2, and HO-1 proteins, culminating in increments to 0.83 ng/ml, 28.28 pg/ml, and 0.89 ng/ml, respectively related to the T/D group.

No statistically discernible variance detected between the sham and eprosartan group. Intriguingly, the elevation in the expression of SIRT1, Nrf2, and HO-1 testicular contents exhibited a heightened prominence in T/D rats subjected to a 60 mg/kg dosage of eprosartan, as contrasted with those receiving a 30 mg/kg dose, as depicted in Fig. 5. These empirical outcomes posit that eprosartan possesses the capacity to incite the activation of the SIRT1/Nrf2/HO-1 axis in T/D rats.

### Eprosartan augmented testicular autophagy by elevating Beclin-1 and attenuating SQSTM-1/p62 expressions in testicular T/D rats

**Figure 7:**
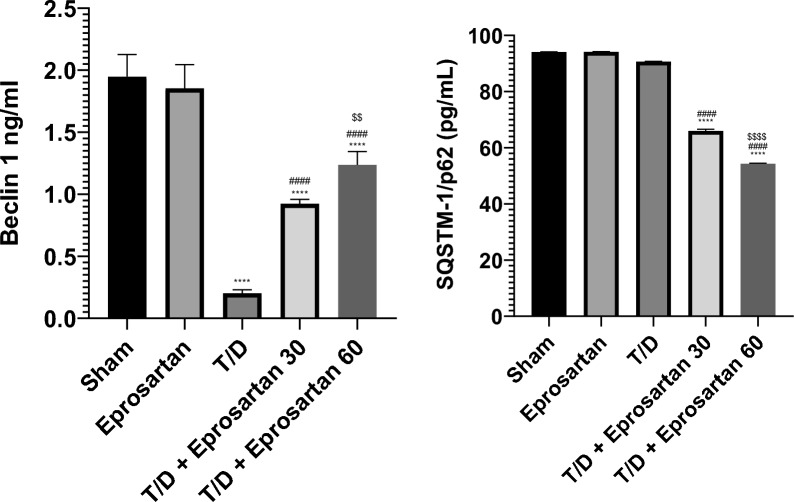
Eprosartan upregulated testicular Beclin-1 and attenuated SQSTM-1/p62 proteins expression across all experimental groups. Statistical analyses were performed employing one-way ANOVA followed by Tukey’s post hoc test, n = 6, mean ± SD. where ****; *p* < 0.0001 related to the sham group, ####; *p* < 0.0001 related to the T/D group, $$$$; *p* < 0.0001, $$; *p* < 0.01 related to the T/D + eprosartan 30 group.

Herein, we delved into the inquiry of whether the therapeutic benefits of eprosartan were intertwined with autophagy. Hence, our investigation encompassed the scrutiny of SQSTM-1/p62 and Beclin-1 proteins expression, as a dependable affirmative hallmark of the autophagic process^25^. As demonstrated in Fig. 7, compared to the T/D group, the administration of eprosartan, either at 30 or 60 mg/kg, effectively activated autophagy, as evidenced by a notable augmentation (*p* < 0.0001) in Beclin-1 protein expression and a notable diminish (*p* < 0.0001) in SQSTM-1/p62 protein expression. This increase in Beclin-1 amounting to 0.92 ng/ml and 1.23 ng/ml, respectively, while SQSTM-1/p62 diminished to 66 pg/ml and 54.3 pg/ml occurred in a dose-dependent fashion. No substantial disparity emerged between sham and eprosartan-treated groups. Remarkably, the pro-autophagic impact of eprosartan at a 60 mg/kg dosage was notably more robust (*p* < 0.01) related to the effect of 30 mg/kg eprosartan treatment in T/D-induced rats. Collectively, these revelations underscore that the capacity of eprosartan to incite testicular autophagy assumes a pivotal role in mitigating the deleterious effects of testicular IRI.

### Eprosartan activates the autophagy-linked AMPK/mTOR pathway in testicular T/D rats

**Figure 8:**
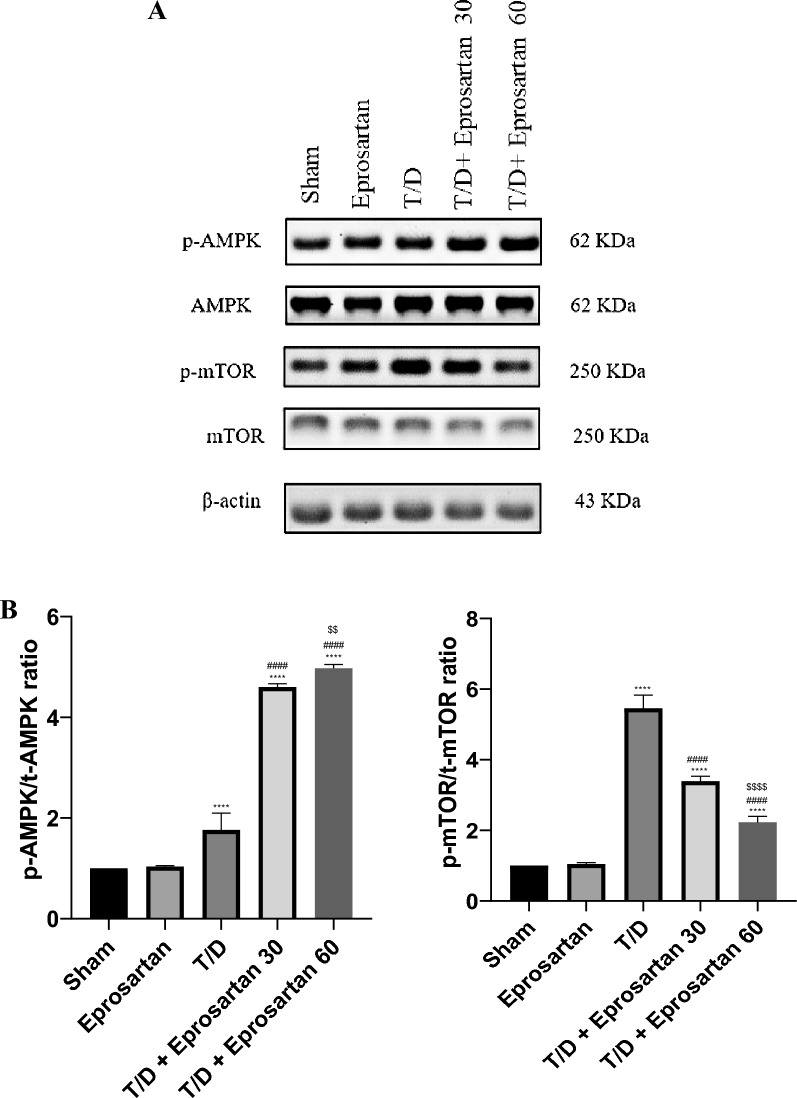
The impact of eprosartan on AMPK and mTOR proteins expression. (**A**) Representative Western blots depicting the phosphorylated and total AMPK, mTOR and β-actin proteins across various experimental groups. (**B**) Expressions of phosphorylated/total ratio of proteins were densitometrically represented as fold change, using bands in (A), relative to that of the sham rats, after normalization to the corresponding β-actin. Bars represent mean ± SD. Followed by Tukey’s post hoc test for multiple comparison, significant difference was analyzed by one-way ANOVA test, where ****; *p* < 0.0001, compared to the sham group, and #### *p* < 0.0001, compared to the T/D group, $$$$; *p* < 0.0001, $$; *p* < 0.01, compared to the T/D + eprosartan 30 group.

We scrutinized the p-AMPK (Ser487)/t-AMPK and p-mTOR (Ser2448)/t-mTOR signaling pathway. Related to the sham group, the AMPK/mTOR pathway within the T/D rat model was decidedly suppressed. This was conspicuously manifested by a moderate elevation in the p-AMPK (Ser487)/total AMPK ratio, and augmentation in the p-mTOR (Ser2448)/total mTOR ratio (*p* < 0.0001), by 5.45-fold change (Fig. 8). However, eprosartan administration at a dose of 30 or 60 mg/kg reversed these alterations and activated AMPK/mTOR pathway, which was observed by a substantial rise in the p-AMPK (Ser487)/t-AMPK ratio (*p* < 0.0001) by 4.59-fold and 4.97-fold increase, respectively, in a dose-dependent approach related to the T/D group. Additionally, there was a substantial reduction in the p-mTOR (Ser2448)/total mTOR ratio (*p* < 0.0001) by 3.38-fold and 2.22-fold decrease following eprosartan administration related to the T/D group.

No substantial disparity was observed between the sham group and the group administered with eprosartan. It is noteworthy that the pro-autophagic impact of eprosartan at a dosage of 60 mg/kg exhibited a significantly higher potency compared to the effect observed with 30 mg/kg eprosartan treatment in T/D induced rats (p-AMPK/t-AMPK (*p* < 0.01) and p-mTOR/t-mTOR (*p* < 0.0001)). Overall, these observations illustrate that the ability of eprosartan to elicit testicular AMPK/mTOR activation plays a role in mitigating testicular IRI.

### Eprosartan reduces cleaved caspase-3 in testicular T/D rats

**Figure 9:**
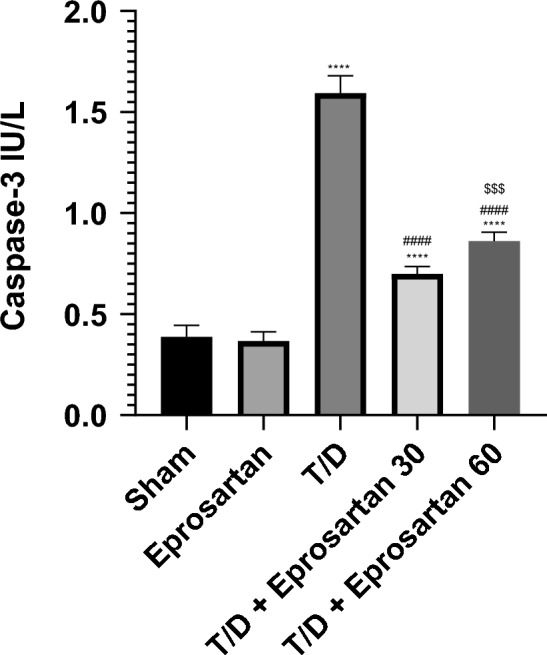
Eprosartan suppressed testicular cleaved caspase-3 across all experimental groups. Statistical analyses were performed employing one-way ANOVA followed by Tukey’s post hoc test, n = 6, mean ± SD. where ****; *p* < 0.0001 related to the sham group, ####; *p* < 0.0001 related to the T/D group, $$$; *p* < 0.001 related to the T/D + eprosartan 30 group.

Within the realm of testicular pathologies, it has been established that hindering the autophagy flux is concomitant with the activation of the pro-apoptotic mechanism^29,57^. Thus, an exploration into testicular apoptosis ensued, entailing the quantification of cleaved caspase-3. Related to the sham group, the testicular tissues of rats afflicted by T/D prominently exhibited a substantial elevation in cleaved caspase-3 (*p* < 0.0001), culminating in an increase of 1.59 IU/L (Fig. 9). Conversely, the administration of eprosartan at dosages of 30 or 60 mg/kg effectively hindered the activation of the apoptotic machinery, distinctly elucidated by a substantial decrement in cleaved caspase-3 (*p* < 0.0001), compared to the T/D-exposed group. This reduction amounted to 0.69 IU/L and 0.83 IU/L, respectively, in a manner reliant upon dosage. Of particular note, no statistically discernible discrepancy emerged between the sham and eprosartan-administered groups.

### Eprosartan suppresses apoptosis in testicular T/D rats

**Figure 10:**
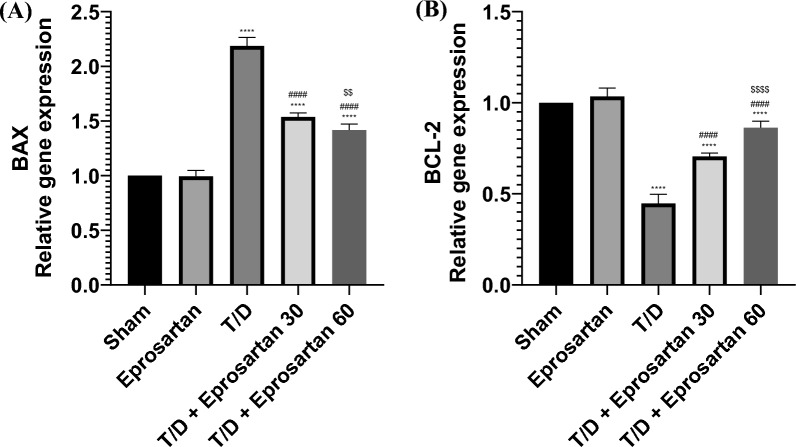
(**A**) Eprosartan downregulated testicular *Bax* gene expression across all experimental groups. (**B**) Eprosartan upregulated testicular *Bcl-2* gene expression across all experimental groups. Statistical analyses were performed employing one-way ANOVA followed by Tukey’s post hoc test, n = 6, mean ± SD. where ****; *p* < 0.0001 related to the sham group, ####; *p* < 0.0001 related to the T/D group, $$$$; *p* < 0.0001, $$; *p* < 0.01 related to the T/D + eprosartan 30 group.

T/D caused a noteworthy increase in *Bax* gene expression (*p* < 0.0001), as well as a noteworthy decrease in *Bcl-2* gene expression (*p* < 0.0001), related to the sham group (Fig. 10). In tandem, the therapeutic intervention in T/D-induced rats through varying dosages of eprosartan (30 or 60 mg/kg) induced a pronounced quelling of *Bax* gene expression (*p* < 0.0001), simultaneously fostering an upswing in *Bcl-2* gene expression (*p* < 0.0001) in comparison to the T/D group. Significantly noteworthy is the observation that the restoration of genes expression of *Bax* and *Bcl-2*, brought forth by the administration of high eprosartan dose in the T/D-induced rats, exceeded that accomplished by the low eprosartan dose in the T/D-induced rats. Between the sham and eprosartan-treated groups, no statistically discernible distinctions were observed. Taken together, these revelations collectively underline the capacity of eprosartan to mitigate testicular apoptosis, elucidating, at least partially, its role in mitigating the deleterious effects imposed upon the testicular milieu in T/D-induced rat models.

## Discussion

Testicular torsion, also referred to as spermatic cord torsion, constitutes a paramount urological emergency^58^. Although the primary intervention involves surgical detorsion of the affected testis, it is crucial to acknowledge that the excessive release of ROS may lead to IRI, contributing to the potential deterioration of testicular tissue^59^. The prospects of testicular salvage exhibit success rates soaring between 90 and 100% when surgical intervention is expeditiously initiated within six hours. However, these chances is significantly diminished to 50% for cases persisting beyond 12 h, and precipitously plummet to sub-10% levels for presentations exceeding a 24-h symptomatic duration^2^.

Autophagy stands as an indispensable guardian of cellular equilibrium, representing a catabolic pathway that removes misfolded proteins and compromised organelles from distressed cells. This orchestrated process unfolds within the autophagy-lysosome pathway, wherein impaired organelles and proteins find their way to the lysosome for decisive dismantling^15,16,18^. In perspective, the elimination of damaged mitochondria represent a pivotal event in mitigating oxidative stress and preserving cellular homeostasis^28^. Of note, autophagy represents a pivotal regulator of IRI, serving vital functions during ischemia reperfusion^60^. Diverse studies illustrate the dual nature of autophagy in IRI. Autophagy is activated in due to energy deficiency, starvation or cellular stress. Promoting autophagic activity within moderate range may offsets mitochondrial damage and aids in maintaining proteostasis in IRI^61^. Nonetheless, certain studies highlight a substantial decline in autophagic clearance during reperfusion, presenting a grave risk to cell survival^62^. Hence, reinstating disrupted autophagic flux could be an effective approach for mitigating IRI. The present study conclusively proves, for the first time, that administration of eprosartan possesses a remarkable capacity to alleviate IRI. This was achieved by instigating testicular autophagy while concurrently quelling apoptotic cellular demise and oxidative phenomena.

In the current study, the discerned autophagic activation likely plays a pivotal role in the augmentation of serum testosterone levels induced by eprosartan. The correlation between testosterone synthesis and testicular autophagy is well-established, as prior research has underscored autophagy's role in fostering testosterone production in Leydig cells by augmenting cholesterol uptake, thereby favoring steroidogenesis^63^. In essence, the intricate interplay between the lysosome system and autophagosome-mediated cholesterol transport significantly bolsters the efficient delivery of cholesterol for steroidogenesis^63,64^. Additionally, the observed reduction in testicular oxidative stress, a probable consequence of eprosartan, is likely instigated by autophagy-mediated eradication of damaged mitochondrial ROS, potentially reinstating steroidogenic enzymes and thus amplifying testosterone synthesis^25^. In addition, the sperm analysis confirmed the impaired spermatogenesis in T/D rats as evidenced by decreased sperm count, motility, viability along with increasing sperm abnormalities. Astonishingly, the administration of eprosartan has restored sperm count, viability, and motility, hence boosted spermatogenesis.

Moreover, T/D rats displayed a substantial reduction in their relative testicular weight, a commonly assessed metric for sperm production^65^. The histopathological analysis revealed distorted seminiferous tubules. This distortion was in the form of reduction of their germinal cells. Distorted interstitial cells of Leydig and widened interstitial were clearly noticed confirming a decline in sperm quality in T/D rats which is aligning with previous studies^29^. In contrast, eprosartan pretreatment reinstated the lost relative testicular weight in addition to a notable enhancement in the structural integrity of the tissue in a dose-dependent manner.

Oxidative stress, primarily impacting germ cells, spermatozoa, and Leydig cells, is intricately linked with the pathogenesis of IRI induced by testicular T/D^66,67^, which harmonizes with the present study's findings as demonstrated by elevated testicular MDA level and exhausted GSH level. Upon detorsion, reperfusion gives rise to escalated lipid peroxidation and germ cells apoptosis^68,69^. While restoring blood flow to ischemic tissue is crucial for its revival, the simultaneous release of free radicals can lead to oxidative damage in cellular membranes, crucial elements, and DNA culminating in inflammation and cell apoptosis^70,71^. Sperms are uniquely susceptible to deterioration by free radicals due to their elevated content of polyunsaturated fatty acids in plasma membranes^72^. Fortunately, administration of eprosartan exhibited a robust shield against oxidative stress as illustrated by decreased MDA level and augmented GSH level highlighting the antioxidant activity of eprosartan. Similar antioxidant activity of eprosartan was elucidated in animal model of diabetic nephropathy^43^. Our findings harmonize seamlessly with antecedent research that showcased eprosartan’s remarkable capacity in alleviating oxidative stress subsequent to IRI^37,44^.

Moreover, the current investigation has elucidated that testicular IRI instigated a deactivation of the SIRT1/Nrf2/HO-1 cascade within the rat testicular tissue. In a broader context, SIRT1 is perceived to occupy a pivotal role in spermatogenesis, deftly modulating male germ cells, Leydig cells, and Sertoli cells, a fact validated in transgenic mouse models^73^. The auspicious influence attributed to SIRT1 stem from its adeptness in suppressing oxidative insults, maintaining energy equilibrium, and promoting mitochondrial biogenesis^73^. In respect to oxidative responses, SIRT1 is renowned for its deacetylation and activation of Nrf2, a pivotal antioxidant pathway that regulates the transcription of several key antioxidants, such as HO-1 and GSH. Notably, scientific report has consistently revealed that therapeutic agents capable of bolstering the SIRT1/Nrf2/HO-1 axis have effectively mitigated testicular IRI^74^. Astonishingly, pretreatment with eprosartan stimulated the SIRT1/Nrf2/Ho-1 signaling pathway which was suppressed by the testicular T/D. Our results harmonize with prior studies demonstrating the anti-oxidant properties of eprosartan in modulating SIRT1 pathway in renal IRI^37^.

In the context of autophagy, Beclin-1 plays a pivotal role in orchestrating the formation of autophagosomes during the sequestration phase, thereby spurring the activation of autophagy^17^. Likewise, SQSTM-1/p62 identifies ubiquitinylated proteins to assist in their removal, and it is also broken down during the process of autophagy. Thus, it serves as a negative indicator of autophagy^15,17^. Substantial evidence supports the notion that interventions aimed at augmenting germ cell autophagy possess the capacity to mitigate testicular dysfunction^28^. Of note, eprosartan, in the present study, possesses the capability to stimulate the autophagy machinery as evidenced by the increased expression of Beclin-1 along with diminishing SQSTM1/p62 accumulation. Interestingly, Autophagy stimulation has previously been identified as a fundamental mechanism by which angiotensin receptor blockers (ARB) mitigate hepatic steatosis^75^, and gastric injury^76^.

Moreover, the current outcomes have unveiled a conspicuous diminish of the testicular AMPK/mTOR pathway, a pivotal autophagy regulator, in response to testicular T/D. This attenuation was evidenced by the heightened expression of p-mTOR. Correspondingly, earlier investigations have illuminated the suppression of AMPK/mTOR in the context of lipopolysaccharide-induced Leydig cell injury in vitro^25^ as well as in experimental models of testicular injury^17^. This is further supported by the recognition that the low energy sensor AMPK is adept at maintaining cellular energy production by initiating autophagy processes via the modulation of the p-mTOR/t-mTOR ratio^25^. AMPK has been recognized as a controller of the quality and functionality of spermatozoa wielding influence on spermatozoa motility, acrosome reaction, and somatic cells’ proliferation^77^. Additionally, the activation of mTOR potentially exacerbates cellular demise by suppressing the protective mechanism of autophagy in ischemic tissues^78^. A recent investigation observed that the instigation of testicular torsion led to heightened mTOR phosphorylation within the seminiferous tubules, instigating oxidative stress, germ cell apoptosis, and subsequent impairment of spermatogenesis. Notably, it was informed that reducing activated mTOR mitigate apoptosis subsequent to IRI in the rat testis^79^. Aligned with these findings, eprosartan effectively invigorated the testicular AMPK/mTOR pathway, as illustrated by the augmented p-AMPK/t-AMPK ratio and the diminished p-mTOR/t-mTOR ratio, thereby stimulating autophagy activation. In consistence with earlier report, it has been observed that the activation of the AMPK/mTOR pathway serves as an underlying mechanism by which ARBs mitigate gastric injury and curtail lipid deposition^76,80^.

Emerging evidence has elucidated the intricate interplay between autophagy flux and the SIRT1/Nrf2/HO-1 pathway. Studies have illustrated that the activation of the SIRT1/Nrf2/HO-1 pathway can impede mTOR signaling, thereby alleviating its inhibitory effects on autophagy^81^. SIRT1 has been observed to deacetylate and activate crucial proteins such as Atg5 (autophagy-related gene 5) and Atg7 (autophagy-related gene 7), which play vital roles in initiating autophagy and facilitating vesicle formation^82,83^. Nrf2, on the other hand, has been informed to promote the transcription of numerous autophagy-related genes, including LAMP2A, SQSTM-1/p62, Atg3, Atg5, and Atg7^84^. Additionally, HO-1 can modulate autophagy by influencing the levels of ROS and maintaining cellular redox balance, both of which are known to impact autophagic processes^85^.

**Figure 11:**
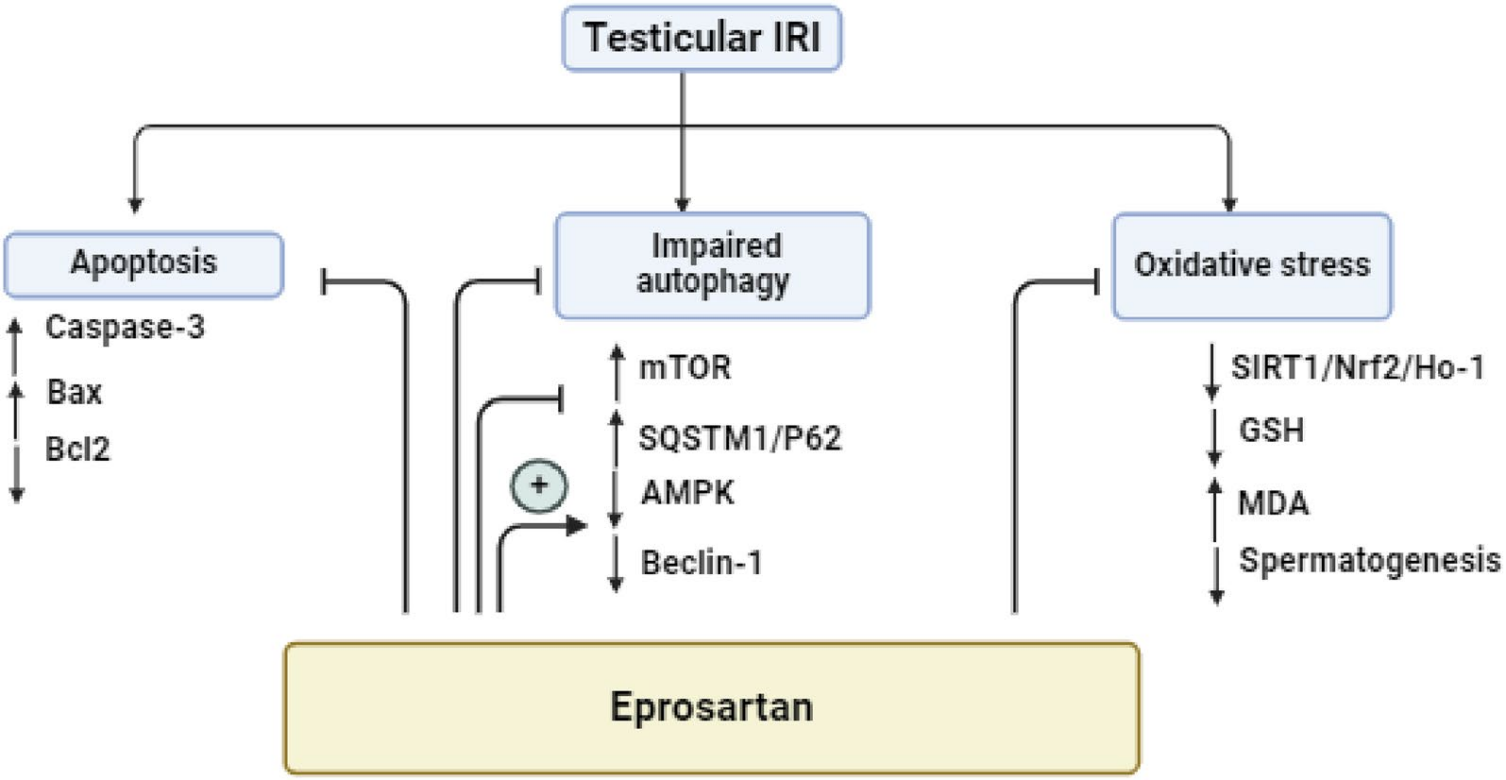
A brief schematic depicting the influence of eprosartan in alleviating testicular dysfunction induced by testicular torsion.

Impaired autophagy culminates in the intracellular accrual of ROS/injured mitochondria, thereby triggering the initiation of apoptotic pathways leading to cell demise^77^. The latter event is characterized by the activation of caspase-3, a process that has been documented to impede the initiation of autophagy by means of caspase-3-mediated degradation of Beclin-1^86^. IRI induced by T/D has been elucidated to predominantly trigger testicular apoptosis, primarily through the mitochondrial pathway^87,88^. The integrity of the mitochondrial membrane is contingent upon the delicate balance between pro-apoptotic Bax and anti-apoptotic Bcl-2 proteins^89^. This alignment with prior report underscores the established correlation between oxidative stress and the induction of apoptotic cell death which is linked to perturbation of spermatogenesis and the integrity of testicular junction proteins^90,91^. Consistent with these observations, the present study unveils that IRI incites pro-apoptotic events in the testis, evident in the heightened expression of *Bax* gene alongside the diminished expression of *Bcl-2* gene, ultimately culminating in the activation of caspase-3 as reflected by the increased cleaved caspase-3 content in T/D rats. On the other hand, eprosartan effectively thwarted the pro-apoptotic processes, evident in the elevation of *Bcl-2*, the suppression of *Bax*, and the attenuation of cleaved caspase-3 testicular content. These favorable incidents are intricately linked to the reinforcement of autophagy mechanisms and the proliferation of spermatogonial cells^92^. Our findings harmonize with earlier research that underscores the concurrent modulation of the apoptotic response and the activation of autophagy induced by ARBs^76^. Overall, eprosartan alleviated the testicular dysfunction induced by testicular torsion as demonstrated in Fig. 11.

## Conclusion

The present study unveils the eprosartan’s remarkable potential in alleviating testicular IRI and rejuvenating impaired spermatogenesis in rats. These auspicious effects stem from eprosartan’s multifaceted activities, encompassing antioxidant, anti-apoptotic, and pro-autophagic mechanisms, via the activation of SIRT1/Nrf2/HO-1 and Beclin-1/SQSTM-1/p62/AMPK/mTOR pathways. These findings propose the prospect of employing eprosartan as an adjunctive strategy to alleviate testicular dysfunction arising from testicular torsion. Nonetheless, further research is warranted to elucidate the additional intricate molecular pathways behind eprosartan's activities.

### Supplementary Information

Supplementary Information.

## Data Availability

Data is provided within the manuscript or supplementary information files.
