# Supplementary figures and images for: Activation of SIRT1/Nrf2/HO-1 and Beclin-1/AMPK/mTOR autophagy pathways by eprosartan ameliorates testicular dysfunction induced by testicular torsion in rats
Source: Sci Rep. 2024 May 31;14:12566. doi: 10.1038/s41598-024-62740-6 (PMC11143266; doi:10.1038/s41598-024-62740-6)

## p-AMPK

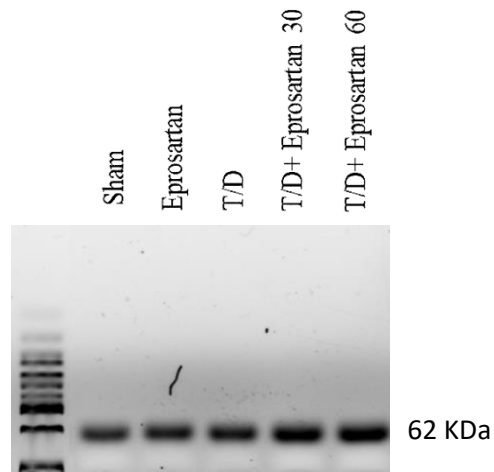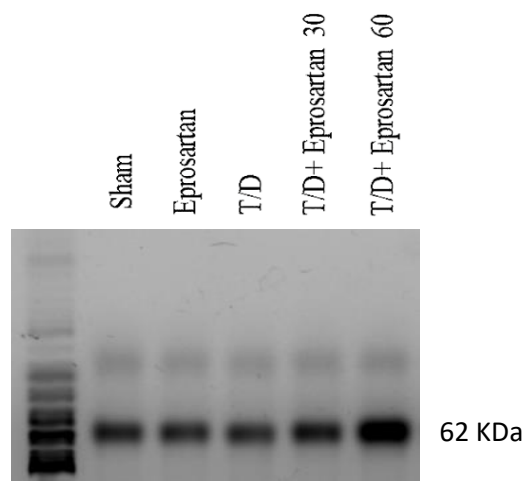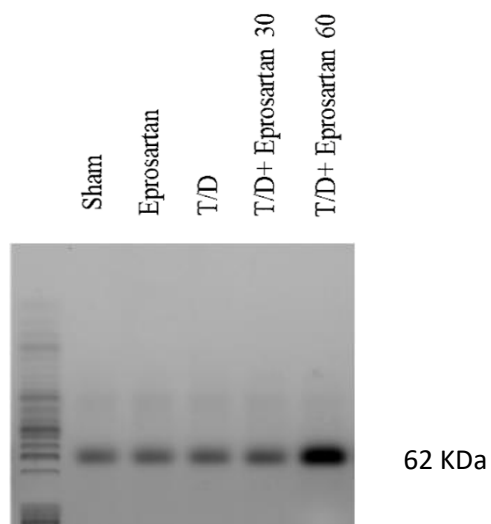

## AMPK

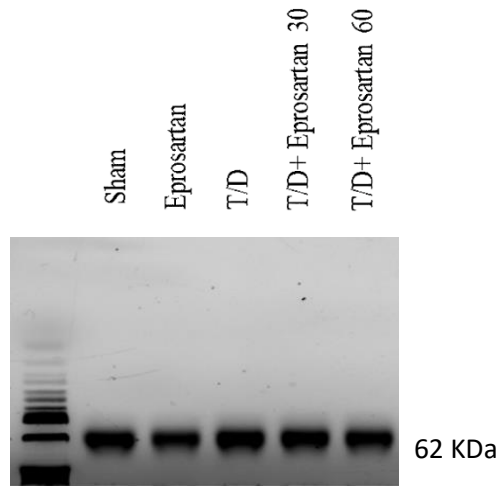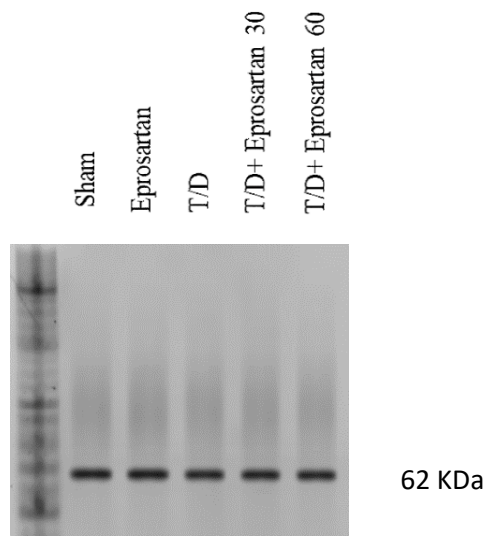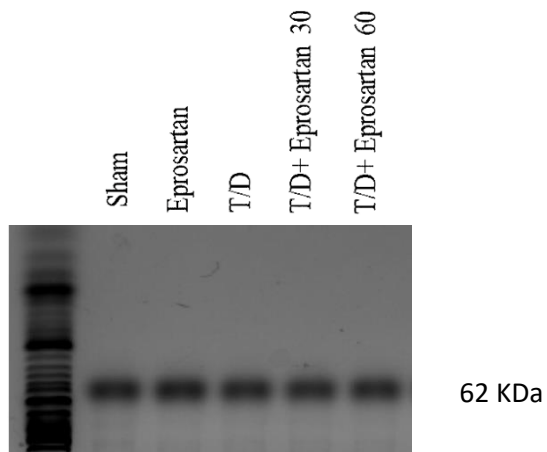

## P-mTOR

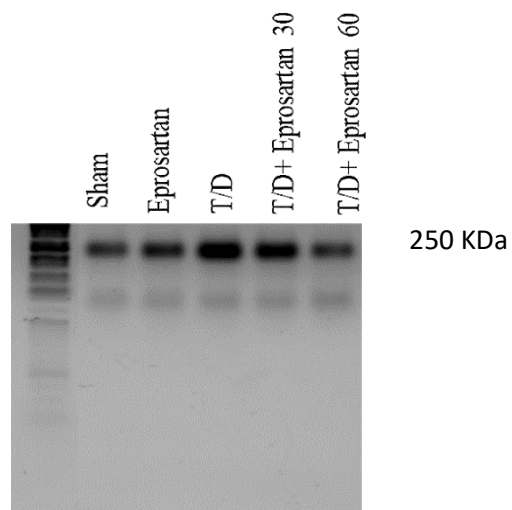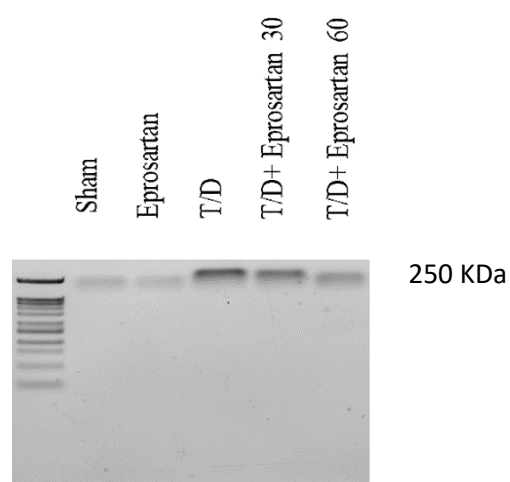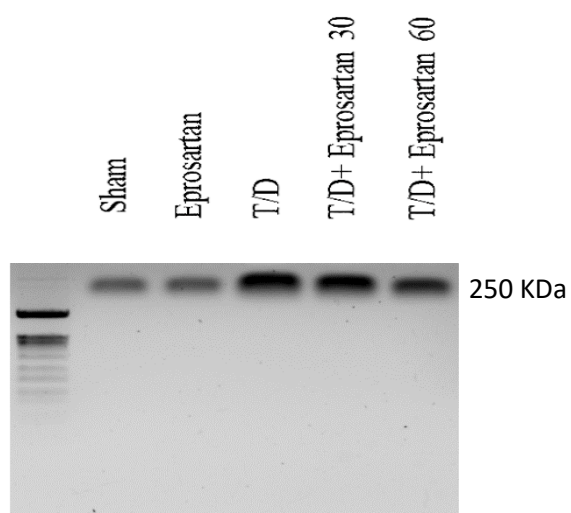

## mTOR

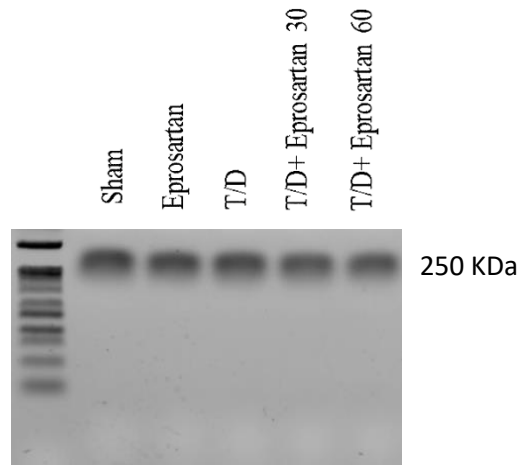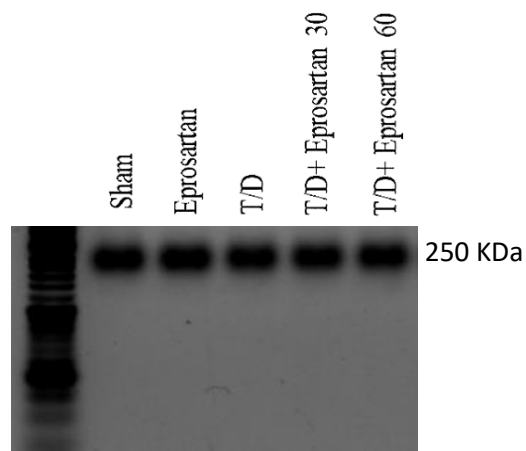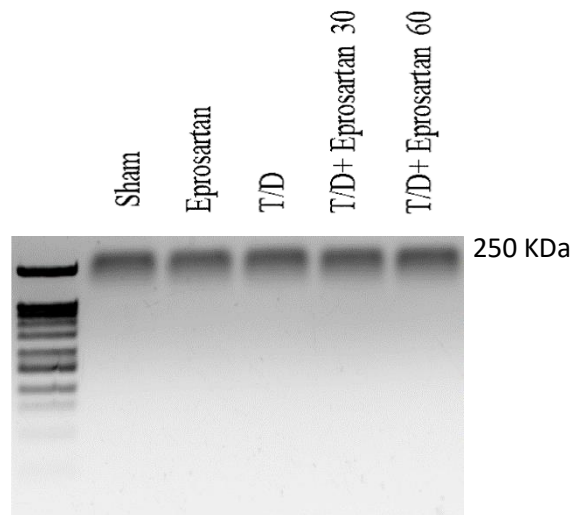

## $\beta$ -actin

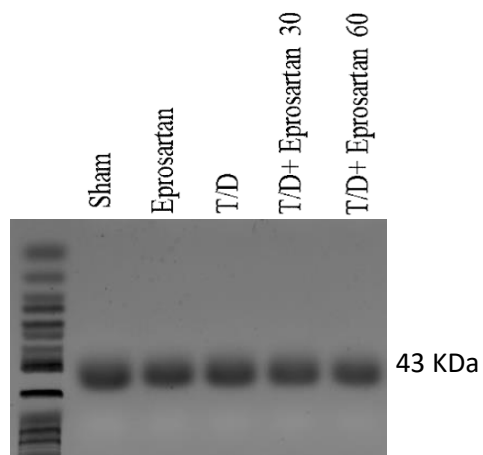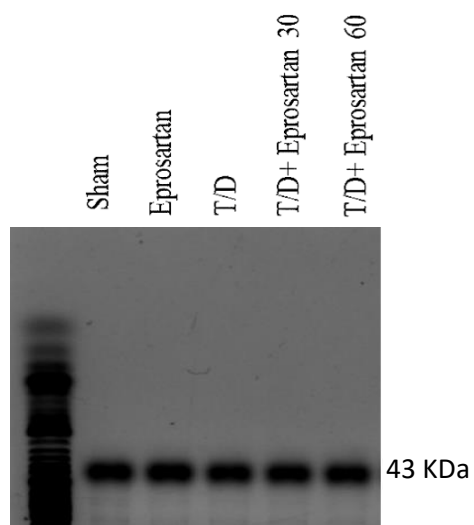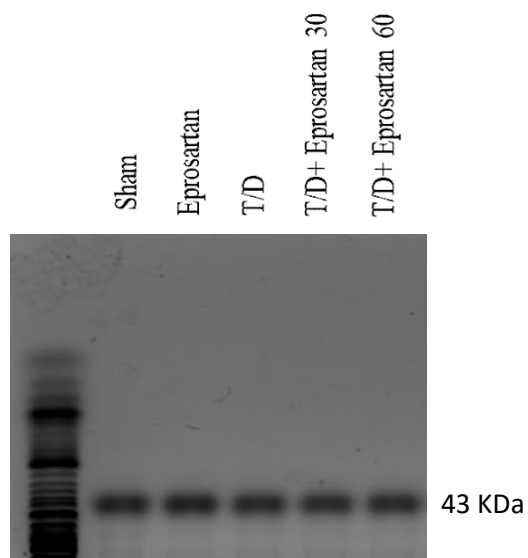

Supplement: Supplementary file 1 — Supplementary Information. [file 41598_2024_62740_MOESM1_ESM.pdf]
